# Co-evolution in a landrace meta-population: two closely related pathogens interacting with the same host can lead to different adaptive outcomes

**DOI:** 10.1038/srep12834

**Published:** 2015-08-07

**Authors:** Domenico Rau, Monica Rodriguez, Maria Leonarda Murgia, Virgilio Balmas, Elena Bitocchi, Elisa Bellucci, Laura Nanni, Giovanna Attene, Roberto Papa

**Affiliations:** 1 Sezione di Agronomia, Coltivazioni Erbacee e Genetica, Dipartimento di Agraria, Università degli Studi di Sassari, Sassari, Italy; 2 Sezione di Patologia Vegetale ed Entomologia, Dipartimento di Agraria, Università degli Studi di Sassari, Sassari, Italy; 3 Dipartimento di Scienze Agrarie, Alimentari ed Ambientali, Università Politecnica delle Marche, Ancona, Italy

## Abstract

We examined the local adaptation patterns in a system comprising several interconnected heterogeneous plant populations from which populations of two phylogenetically closely related pathogens were also sampled. The host is *Hordeum vulgare* (cultivated barley); the pathogens are *Pyrenophora teres* f. *teres* (net form) and *Pyrenophora teres* f. *maculata* (spot form), the causal agents of barley net blotch. We integrated two approaches, the comparison between the population structures of the host and the pathogens, and a cross-inoculation test. We demonstrated that two closely related pathogens with very similar niche specialisation and life-styles can give rise to different co-evolutionary outcomes on the same host. Indeed, we detected local adaptation for the net form of the pathogen but not for the spot form. We also provided evidence that an *a-priori* well-known resistance quantitative-trait-locus on barley chromosome 6H is involved in the co-evolutionary ‘arms race’ between the plant and the net-form pathogen. Moreover, data suggested latitudinal clines of host resistance and that different ecological conditions can result in differential selective pressures at different sites. Our data are of interest for *on-farm* conservation of plant genetic resources, as also in establishing efficient breeding programs and strategies for deployment of resistance genes of *P. teres*.

Understanding the contributions of different evolutionary forces in the shaping of population biodiversity is a relevant goal in evolutionary genetics, as it addresses the relative importance of neutral *versus* adaptive processes. Moreover, this can allow strengthening of the scientific basis for correct management of genetic resources, and identification of sources of genes for adaptation.

In general, local adaptation is estimated by comparison of the fitness of populations in their own habitat with that observed in other habitats. More in particular, unlike adaptation to the physical environment, adaptation to another species can induce reciprocal genetic responses, whereby the host evolves to decrease the effectiveness of the adaptation of a pathogen, and the pathogen evolves to decrease the effectiveness of the defences of the host^1^. In this context, the two players give rise to the so-called co-evolutionary ‘arms race’^2^. Here, pathogens are said to be locally adapted if their virulence on sympatric host populations is higher compared with the allopatric host populations. On the other hand, the host is locally adapted if it is more resistant to sympatric than allopatric pathogens^3^. Local adaptation depends on several factors, such as the genetic basis of the adaptation of the host and the pathogen, the gene flow among the local demes, each of which has its own selective history, and several other life-history traits such as generation time and mating system^4^,^5^,^6^. However, a meta-analysis revealed that the pathogen is more often locally adapted to the host, albeit that cases of pathogen maladaptation (i.e., of pathogen that are less able to infect local host) have also been documented^7^.

To understand if there is the potential for a host and a pathogen to become locally adapted, it is useful to compare the population structures of both of these partners^8^. The rationale followed in these studies is that when the organisation of genetic variation does not match up between the two protagonists, stochastic forces rather than co-evolution are believed to be responsible for the maintenance of resistance and the virulence diversity^8^. On the contrary, a parallelism in the population structures of both the plant and the pathogen can be an indicator of co-evolution processes^8^. The use of molecular markers in such investigations has been extensive. Several studies have used isozymes, *random-amplified polymorphic DNA*, *restriction fragment length polymorphism,* and *simple sequence repeats* (SSRs) to compare host and pathogen population structures in various types of interactions, such as plant/insect^9^, and plant/parasitic plant^10^,^11^ interactions. Considering plant/fungi interactions, for example, stochastic forces were suggested for the *Linum marginale*/*Melampsora lini* interaction^12^, while co-evolution was suggested for the *Phaseolus vulgaris*/*Colletothricum lindemunthianum* interaction in central and southern America^8^. However, other than by co-adaptation, the same population structure for two protagonists can result from similar migration/drift dynamics, or from parallel environmental pressures (e.g., climate, edaphic) acting upon the two protagonists^8^.

A second, more direct, approach used to investigate patterns of local adaptation is to inoculate a plant with a pathogen. In this case, local adaptation has generally been tested in two ways: (1) by checking the pathogen performance on sympatric *versus* allopatric hosts (‘home vs away’); and (2) by checking sympatric *versus* allopatric pathogens on a given host population (‘local vs foreign’)^9^. However, variations in host resistance can obscure tests using the former method, while variations in pathogen virulence can obscure the latter^16^,^17^. Thus, experimental tests that control simultaneously for variations in both plant and pathogen populations in a reciprocal cross-inoculation design are preferable^12^,^13^.

Local adaptation studies are more frequently conducted in wild ‘undisturbed’ contexts. Notwithstanding this, studies conducted in agricultural systems can be particularly relevant, as resistance to diseases and pests are characters of widespread use and are of paramount importance in plant breeding. When working with cultivated species, landraces offer a unique opportunity. Indeed, landraces are populations of cultivated species that have evolved under stochastic and demographic processes (mutation, drift, migration), and also under human-mediated and natural selection. Moreover, landraces are often characterised by high levels of genetic variability^14^,^15^ and have an ancient link with the environment in which they are cultivated. In this context, investigations have included the scale of the patterns of variation (e.g., for *Puccinia arachidis* and *Phaeisariopsis personata* with *Arachis hypogea*^16^) and the relative importance of major, race-specific resistance and the relationships between reactions to exotic *versus* endemic pathotypes (e.g., for *Hordeum vulgare* and its two major pathogens *Puccinia hordei* and *Blumeria graminis*^17^,^18^). However, comprehensive studies conducted with landraces at the meta-population level are lacking.

In the present study, we investigated local adaptation in the context of a barley landrace meta-population in terms of the *Hordeum vulgare* L.—*Pyrenophora teres* plant–pathosystem.

The host plant was the cultivated barley, *H. vulgare*, which is one of the most important crops throughout the world. It is a diploid (2n = 2x = 14) strictly selfing (outcrossing <1%^19^) annual species. In a situation that is almost unique in Europe, along with the cultivation of modern varieties, in Sardinia are still used local six-rowed populations of an old landrace known as *S’orgiu sardu*^20^. Sardinian farmers are not able to discriminate among the local populations, and they use the same name for all of them^20^. These farmers appreciate, maintain and cultivate *S’orgiu sardu* mainly due to its suitability for green fodder production for direct sheep grazing. Moreover, it has been shown that Sardinian barley has a higher yield stability compared to modern varieties when it is grown under highly variable inter-annual meteorological conditions^21^. These populations have been characterised using several types of molecular markers, such as isozymes and *random-amplified polymorphic DNA* markers^22^, *sequence-specific amplifications* polymorphisms (SSAP^15^) and microsatellite (SSRs^23^). Of particular interest is that the populations are highly variable, with most of the individuals analysed having a unique multilocus genotype. Moreover, all of these molecular analyses have also shown relatively low levels of differentiation among the populations. Such landrace populations have also displayed relevant levels of phenotypic variation for key agronomic traits^24^,^25^.

The pathogen was the parasitic ascomycetes fungus *P. teres* Drechsler (anamorph: *Drechslera teres* [Sacc.] Shoemaker). *Pyrenophora teres* is the causal agent of barley net blotch, which is an increasingly damaging foliar disease with a worldwide distribution that can cause substantial yield losses^26^. Two morphologically similar intraspecific *formae speciales* of the pathogen are known. The ‘net’ form (*P. teres* f. *teres; Ptt*) produces elongated, light-brown lesions, with dark-brown necrotic reticulations, while the ‘spot’ form (*P. teres* f. *maculata; Ptm*) produces ovoid, dark-brown lesions that are surrounded by distinct chlorotic areas^27^. Based on amplified fragment length polymorphism (AFLP) and mating-type gene sequence analyses, it has been shown that isolates from infected fields of *S’orgiu sardu* barley landraces can be clearly split into two strongly defined pathogen groups that correspond to the *Ptt* and *Ptm* forms, which indicates that there are two closely related, but reproductively isolated, pathogen groups in Sardinia^28^,^29^. Moreover, the low level of linkage disequilibrium^28^ and the co-existence in each field of both mating types at similar frequencies^30^ strongly suggest that as well as clonal reproduction, sexual reproduction is also likely to be very important in the generation of the observed diversity in both of these *Ptt* and *Ptm* pathogens. Finally, it has also been suggested that drift (or founder effects) and low gene flow might have a more prominent role in the *Ptt* than the *Ptm* pathogen^28^.

Different host genes confer resistance to the *Ptt* and *Ptm* pathogens (e.g. table 1 of^31^) which suggests that there have been separate host—pathogen evolutionary process for these two pathogen forms. Thus, in the present study, we investigated this ‘host—two pathogens’ plant-pathosystem for the local adaptation of the host and/or pathogen. Specifically, we ask the question whether the patterns of variations observed for the *Ptt* and *Ptm* pathogens reflect the same or at least partially independent underlying evolutionary processes.

With these aims, we compared the population genetics structure of the host *H. vulgare* with those of the pathogens *P. teres* f. *teres* and *P. teres* f. *maculata*, and we used two cross-inoculation designs to directly test for the local adaptation, separately for both of the two *formae* of *P. teres*. This allowed us to determine the consistency of different approaches for the detection of local adaptation, and we discuss further the differences between the *Ptt* and *Ptm* pathogens in their co-evolutionary interactions with their host.

## Results

### Neutrality tests

Among the 134 SSAP markers used to characterise the barley populations, six (4.48% of the total) were under divergent selection, with F_ST_ varying from 0.499 to 0.777 (average, 0.601) and with P values between 0.05 and <0.001. Among the AFLPs used to characterise the pathogens, eight markers out of 112 (7.14% of the total) were under selection among the *Ptt* populations of the pathogen, while only two out of 114 (1.75% of the total) were under selection among the *Ptm* populations. The average F_ST_ values of the markers under selection in the *Ptt* and *Ptm* were 0.716 and 0.292, respectively. The P values were between 0.05 and <0.001 for the *Ptt*, and <0.05 for the *Ptm*. From hereafter, when necessary, we present calculations performed on the entire dataset or only using putatively neutral or putatively selected loci.

### Comparison between plant and pathogen population structure for molecular markers

#### Within population diversity

**Figure 1:**
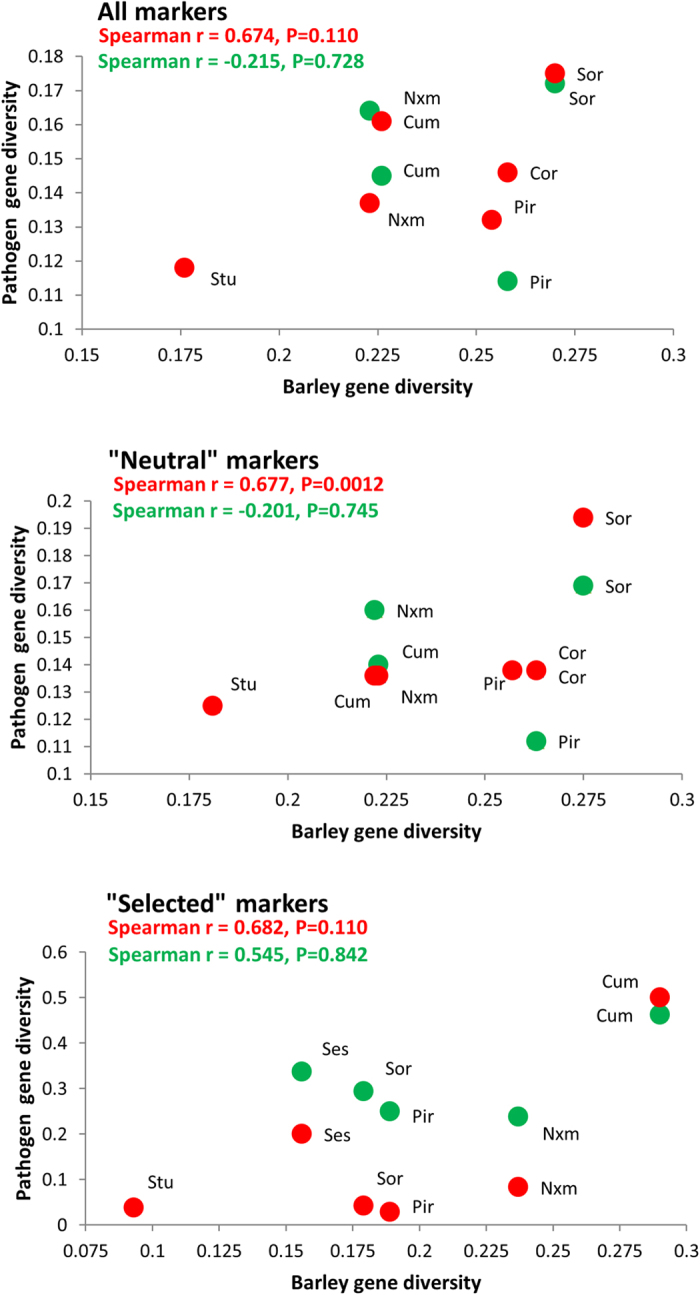
Correlations (Pearson’s ‘r’) between pathogen and host population gene diversity (Nei, 1978). The significance of each correlation is based on the Spearman-rank non-parametric method. Calculations were performed for the *Ptt* (red) and *Ptm* (green) separately, and using all of the markers, only the putatively neutral markers, or the markers putatively under divergent selection.

Considering all of the markers, the genetic diversity of neither the *Ptt* populations nor the *Ptm* populations were significantly correlated with the genetic diversity of the barley host, albeit a stronger effect was noted for the *Ptt* than for the *Ptm* (r = 0.674, P = 0.110 *vs* r = −0.215, P = 0.728; Fig. 1). Interestingly, when only putatively neutral loci were considered, a difference between the *Ptt* and *Ptm* forms emerged: while the diversity of the *Ptt* populations and host populations were strongly correlated (r = 0.677, P = 0.0012), there was no apparent association between the *Ptm* and the barley diversity (r = −0.201, P = 0.745; Fig. 1). When loci putatively under divergent selection were used, the correlations did not reach significance, although a difference between the *Ptt* and *Ptm* forms of the pathogen was still suggested (*Ptt*: r = 0.682, P = 0.110 *vs Ptm*: r = 0.545, P = 0.842; Fig. 1).

#### Between population diversity

Considering all of the markers, simple correlation analysis indicated that the genetic distances between the *Ptt* populations were significantly associated to those between the barley host populations (r = 0.586; P = 0.0107), while this was not the case for the *Ptm* populations (r = −0.079; P = 0.577) (Table 1). There was a similar pattern also for the correlations between the genetic distances and the geographic distances, albeit these did not reach statistical significance (r = 0.700, P = 0.0725 and r = 0.200, P = 0.306 for the *Ptt* and *Ptm*, respectively; Table 1).

The correlation between host population divergence and geographic distance never reached statistical significance; however, positive trends were observed (r = 0.246 and 0.431, respectively; Table 1). Thus, to disentangle the effects of geographic distance from those due to genetic distances between host populations on the pathogen population genetic divergence, a three-way Mantel test was performed to calculate the partial correlations (r) between the pathogen (*Ptt* or *Ptm*) and the host genetic distance, and between the pathogen genetic distance and the geographic distance. This confirmed that the genetic divergence between the barley populations explained a significant (r = 0.597, P = 0.0084) and non-trivial portion of the variance of the pair-wise population divergence for the *Ptt* (25.8%). However, this correlation was not significant for the *Ptm* (r = −0.186, P = 0.6381), with only 1.59% of the variance explained (Table 1). Similarly, the geographic distances tended to have a stronger impact on the *Ptt* population divergence (41.4%) than on the *Ptm* population divergence (5.75%) (Table 1).

The analysis conducted with the loci under selection confirmed that the genetic divergence between the barley populations explained a significant (r = 0.443, P = 0.0028) proportion of the variance of the pair-wise population divergence for the *Ptt* (14.7%). This was not the case for the *Ptm* (r = −0.021, P = 0.433), with only 0.10% of the variance explained (Table 1). The calculations with the neutral dataset also suggested differences, albeit less sharp, between the *Ptt* and *Ptm* forms (25.3%, P = 0.0570; 9.31%, P = 0.2082; respectively). Similarly, the geographic distance tended to have a stronger impact on the *Ptt* population divergence (33.1%–34.7% of the variance explained, depending on the dataset considered) than on the *Ptm* population divergence (0.05%–8.90%) (Table 1).

In Table 2, the mean F_ST_ values are presented for both the host and the pathogens. The genetic divergence among populations was statistically highly significant (AMOVA: P < 0.001 in all cases). Considering the entire marker datasets, the *Ptt* populations (F_ST_ = 0.276) were about four-fold more differentiated than the *Ptm* populations (F_ST_ = 0.071). The F_ST_ obtained for the barley landrace populations (F_ST_ = 0.189) was in between those obtained for the *Ptt* and *Ptm* forms of the pathogen. Thus, the host populations were about 30% less differentiated than those of the Ptt pathogen (F_ST_ = 0.276), and 2.0-fold to 2.5-fold more divergent than those of the *Ptm* pathogen (F_ST_ = 0.071). The Wilcoxon non-parametric-tests indicated significant differences in the mean F_ST_ among the three organisms (P < 0.05), which further resulted in these all being separated based on the Tukey-Kramer tests (Table 2). Moreover, the *Ptt* and *Ptm* forms of the pathogens showed disjoint F_ST_ bootstrap 95% confidence intervals. When the host and the pathogens were compared, the 95% confidence intervals marginally overlapped for the barley and the *Ptt*, and were disjoint for the barley and the *Ptm* (Table 2).

Assuming an island model of population structure, differences in the F_ST_ estimated can be translated into differences in migration rates, mN_isl_ (Table 2). The *Ptm* showed the highest mN_isl_, followed by the barley host and then the *Ptt*. This pattern among these three organisms held also when the ‘neutral’ marker datasets were considered, even if, as expected, the F_ST_ decreased and the estimates of the migration rates consequently increased (Table 2).

### Resistance structure and local adaptation

**Figure 2:**
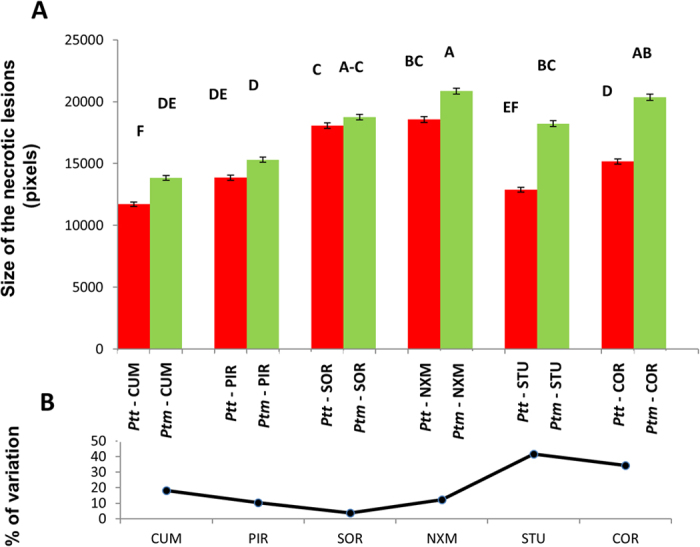
Interactions for the pathogen *forma specialis* × plant population. (**A**) Level of resistance (pathogen aggressivity) that are not connected by the same letters are different, with P < 0.05 based on Tukey-Kramer tests. (**B**) Differences (%) between the performance of the *Ptt* and the *Ptm* on the same host population.

**Figure 3:**
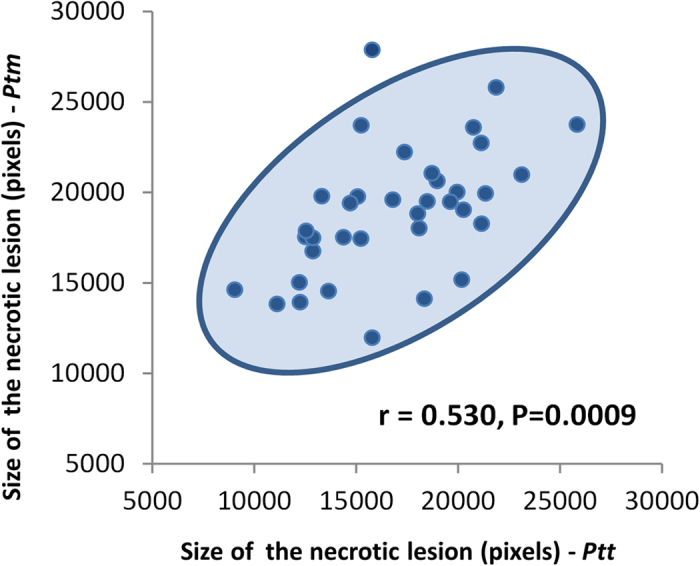
Correlation between size of the necrotic lesions produced by *Ptt* and *Ptm* across the 36 barley lines considered in the present study. The density ellipse (α = 0.95) is also drawn.

The barley fields differed significantly in terms of the distribution of the two *formae speciales* of the pathogen (χ^2^ test, P < 0.001) (Table S1). The strongest difference was observed for CUM, in the north of the island, which showed highly significant prevalence of the *Ptm* (P < 10^−6^), and then for STU, in the south of the island, where in contrast, there was significant prevalence of the *Ptt* (P = 2 × 10^−9^). Thus, we first tested for the significance of the pathogen *forma specialis* × barley populations interaction. Interestingly, ANOVA showed that the barley populations responded differently towards the *Ptt* and *Ptm* forms of the pathogen (Table 3). In particular, four populations (i.e., CUM, NXM, STU and COR) were more susceptible to the *Ptm* than the *Ptt*, while no significant differences where observed for the remaining two populations (PIR and TER) (Fig. 2). Overall, the *Ptm* produced bigger necrotic lesions than the *Ptt* ( + 16.84%; P < 10^−4^). Moreover there was a significant, but not strong, correlation between the host-line susceptibility to *Ptt* and *Ptm* pathogens (r = 0.530, P = 0.0009; Fig. 3).

All of these findings suggested differences among the populations and single individuals in the resistance genes against *Ptt* and *Ptm*. Thus, from here on we considered the results from the cross-inoculation experiments for the *Ptt* and *Ptm* pathogens separately.

**Figure 4:**
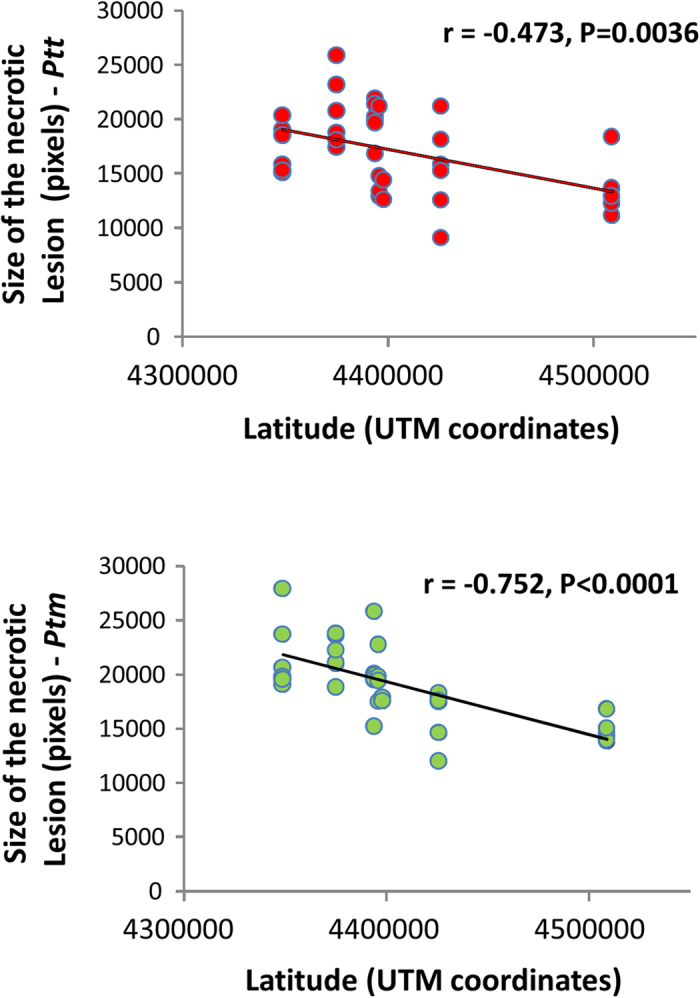
Correlation between size of the necrotic lesions on the barley lines and latitude of provenance. Calculations are given separately for the *Ptt* (top) and the *Ptm* (bottom) of *P. teres*.

Here, a significant variation in the mean resistance among the barley populations was observed, which was true for both the *Ptt* and *Ptm* (Table 4). Moreover, the resistance of the host populations increased when moving from the south to the north of the island; i.e., there was a latitudinal cline in the host genetic resistance (Fig. 4). This was true for both the *Ptt* and *Ptm* pathogens, albeit there was a stronger effect for the *Ptm* (Fig. 4). Although we detected a statistically significant difference in the mean aggressivity among the *Ptt* and *Ptm* pathogen populations (Table 4), there was no associated latitudinal pattern (Figure S1). Moreover, there was no significant correlation between the mean resistance of the host populations and the mean aggressivity of the associated pathogen samples (P > 0.05).

**Figure 5:**
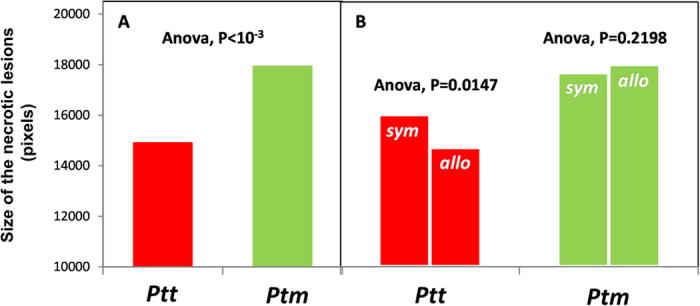
Mean size of the necrotic lesions produced by *Ptt* and *Ptm* (**A**), and separately for the Sympatric (*sym*) *versus* Allopatric (*allo*) population combination within the *Ptt* and the *Ptm* form (**B**).

Interestingly, the ANOVA showed that the Sympatric *versus* Allopatric term was significant for the *Ptt* but not for the *Ptt* form (Table 4). This was seen by analysing the entire dataset (Table 4) or the data from the populations from which the pathogen samples were available (Table S2). In particular, we observed that the *Ptt* isolates produced 8.87% greater lesions in the sympatric interactions than in the allopatric interactions (Fig. 5); this provided evidence that *on average* and *at the meta-population level* the *Ptt* pathogen is locally adapted. Conversely, for the *Ptm*, no local adaptation or maladaptation (i.e. lower ability to infect local host than allopatric host populations) can be inferred.

Jack-knifing over the populations indicated that the significance of the Sympatric *versus* Allopatric term observed for the *Ptt* was mainly due to the CUM and STU populations (Table 5). Indeed, elimination of CUM or STU from the dataset resulted in non-significant P values (Table 5), while elimination of one of the population among the PIR, TER and NXM do not modify the inference of local adaptation. The marked effects of the CUM and STU populations was also confirmed when we analysed the data from the populations from which the pathogen samples were sampled (Table S3). Interestingly, these populations were quite far apart (about 150 km), and as stated above, they were also characterised by very different *Ptt*/*Ptm* ratios (Table S1).

### Comparison between population structure and cross-inoculation studies

**Figure 6:**
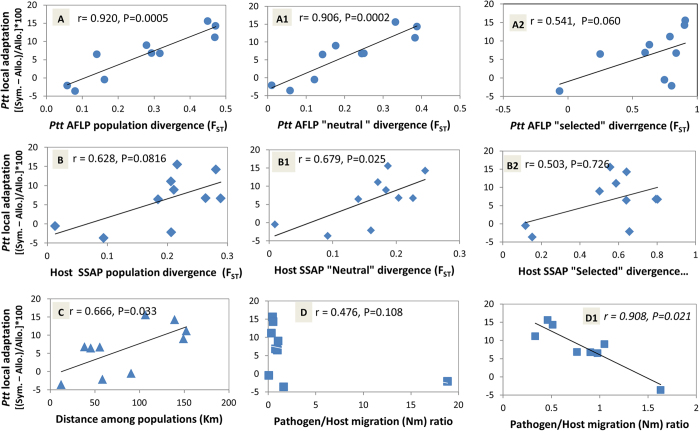
Correlations between the strength of local adaptation of the *Ptt* pathogen and the genetic (F_ST_) and geographic (km) distances between the populations. For each pair of populations, local adaptation was measured by the differences in the sympatric–allopatric comparison using the population least square mean calculated by applying the statistical model in Table 4. Genetic distances between the pathogen populations are based on AFLPs (**A**), and those among the host populations are based on SSAPs (**B**). For each correlation, we report the coefficient of correlation (r), while the P values were determined by the Spearman-rank non-parametric method.

As we showed local adaptation for the *Ptt* pathogen, we further investigated the relationships between the population structure and the local adaptation patterns. Interestingly, the strength of the pathogen local adaptation was positively and very strongly significantly correlated with the pathogen population divergence as measured by AFLPs (r = 0.920, P = 0.0005; Fig. 6A). On the contrary, local adaptation did not significantly depend on the host population divergence when this was measured by all of the SSAPs (r = 0.628; P = 0.0816; Fig. 6B). Furthermore, using the putatively ‘neutral’ markers, the local adaptation was significantly correlated with the population divergence of both the pathogen (r = 0.906; P = 0.0002) and the host (r = 0.679; P = 0.025) (Fig. 6A1), which was not the case when using markers that carried the signature of the divergent selection among the populations, albeit also here there was a slightly stronger effect for the pathogen compared to the host (AFLP: r = 0.541, P = 0.060; SSAP: r = 0.503, P = 0.726; Fig. 6A2). Moreover, the correlation between the local adaptation and geographic distance (r = 0.666, P = 0.033; Fig. 6C) was less strong than the correlations between the local adaptation and the neutral AFLP (Fig. 6A1) or neutral SSAP (Fig. 6B1), which suggested a fundamental role for the population structure of the two players in the co-evolution process.

The ratios between pathogen migration and host migration were not significantly predictive of the Sympatric *versus* Allopatric differences for the pairs of populations (r = 0.476, P = 0.108; Fig. 6D). However, after removing two population pairs for which the population divergence was not significant (one for the pathogen, one for the host; Table S4), the correlation become negative and significant (r = 0.908, P = 0.021; Fig. 6D1), which suggested that in this system there was higher pathogen local adaptation when the pathogen migrated less than the host.

**Figure 7:**
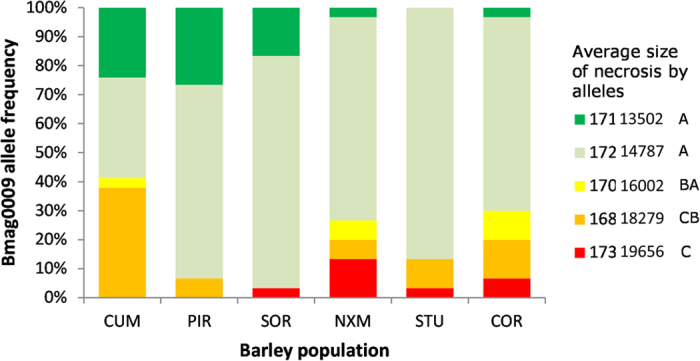
Distribution of the five alleles found at Bmag0009 SSR locus within and among the six Sardinian barley landrace populations. On the right part of the figure it is reported the average size of the necrosis (in pixels) for each of the five alleles. Means that do not share the same letter are different (P < 0.05) based on Tukey-Kramer HD test.

Finally, we tested the hypothesis that a major QTL on chromosome 6H that confers the resistance of barley against the *Ptt* of barley net blotch in several genetic backgrounds^32^,^33^ is responsible (at least partially) for the observed patterns of variation. With this aim, we genotyped the individual barley plants at a microsatellite locus (Bmag0009) that is associated (at a distance < 0.7 cM) to this major resistance QTL^34^. Here, the Bmag0009 locus do not show any signature of divergent selection (P > 0.05). However, the populations showed moderate significant allelic divergence at this locus (AMOVA = average F_ST_ = 0.132; P < 0.0001; Fig. 7. In this regard, it is of note that the CUM and STU populations that are the most divergent at the Bmag0009 locus (F_ST_ = 0.277, P < 0.0001; Fig. 7; Table S4) were also those with the most biased and contrasting *Ptt*/*Ptm* ratios (Table S1) and that produced the strongest signals of local adaptation (Table 5). As expected, the different alleles at Bmag0009 locus are associated to different levels of resistance (Fig. 7). Notably, Fig. 7 also evidenced that moving from North to South the allele associated with the lowest susceptibility decreases in frequency while tend to be more frequent that associated with the highest susceptibility. Additionally, the ANOVA showed that the Bmag0009 genotype × *Ptt* pathogen population interaction was significant (F_20,3391_ = 2.268, P = 0.001), which indicated that there were different effects of the *Ptt* pathogen populations on necrosis size in barley with different Bmag0009 genotypes, or alternatively, that there were different effects of the Bmag0009 genotype on necrosis size in barley infected by the different *Ptt* pathogen populations. In contrast, the interactions for Bmag0009 × pathogen population were not significant for the *Ptm* (F_20,3305_ = 0.601, P = 0.915).

**Figure 8:**
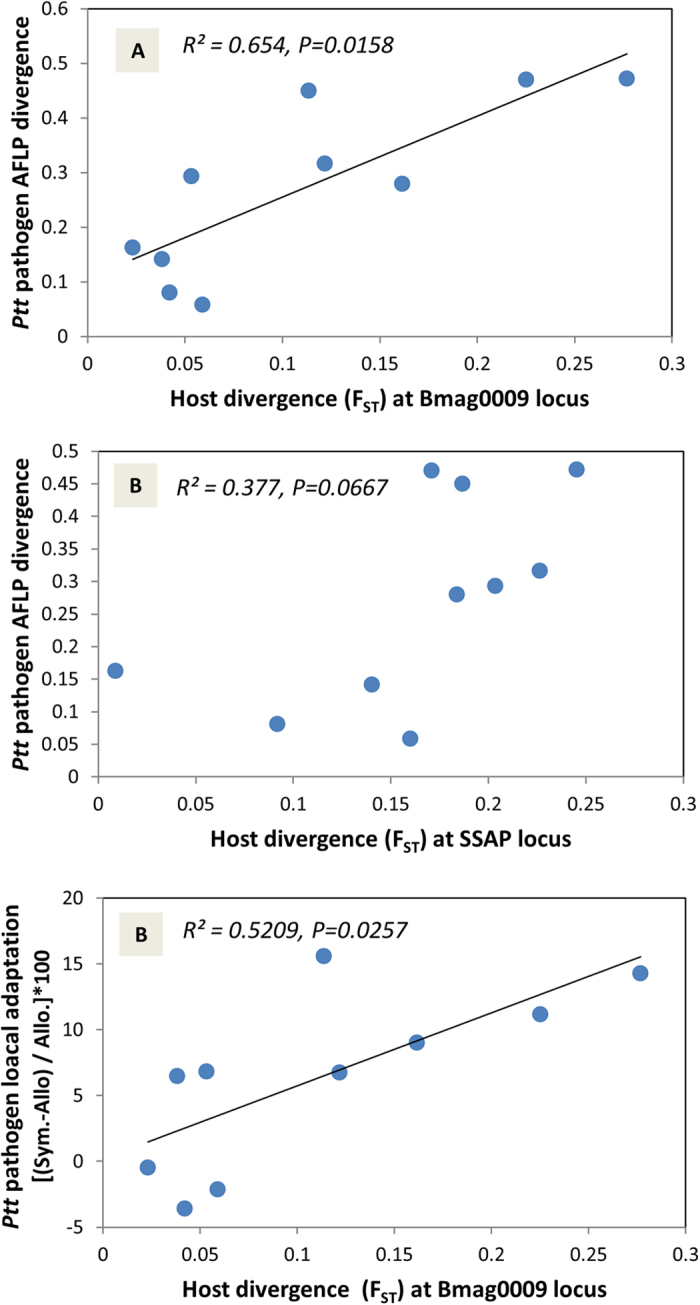
Associations between the *Ptt* population structure and the population divergence (F_ST_) at the Bmag0009 locus (associated to a QTL for *Ptt* resistance) and all of the SSAPs (A, B), and between the *Ptt* local adaptation and the F_ST_ at the Bmag0009 locus (C). The significance of each correlation is based on the Spearman-rank non-parametric method.

Interestingly, the divergence between the *Ptt* populations was more correlated with Bmag0009 (r = 0.809, P = 0.0158; Fig. 8A) than with SSAP (r = 0.614, P = 0.0677; Fig. 8B), which indicated that the *Ptt* population structure better-mirrored the spatial distribution of the locus-specific QTL resistance alleles (linked to Bmag0009) than the multilocus population structure depicted by the anonymous SSAP markers. Furthermore, the local adaptation of the *Ptt* pathogen was best explained by the host population divergence at Bmag0009 (r = 0.722, P = 0.0257; Fig. 8C) than by the host SSAP divergence (r = 0.614, P = 0.0677; Fig. 8B) or the geographic distances (r = 0.666; P = 0.0330; Fig. 6C). Thus, the pathogen populations collected from the host populations that diverged at the Bmag0009 locus (i.e., at the *Ptt* resistance QTLs on chromosome 6H) were also genetically more differentiated and produced stronger signals of pathogen local adaptation.

Collectively, this suggests that the pathogen variation was probably tracking the *Ptt* resistance QTLs on chromosome 6H.

## Discussion

To study the co-evolutionary relationships between barley and *P. teres*, we conducted analyses at the meta-population level, through integration of the comparisons of the population structures of both host and pathogens with the cross-inoculation design.

In this study, the AFLP analyses do not have the main aim of estimating the variability within populations. Moreover our study does not have the aim of comparing differences in allele frequencies among populations at single-locus level, a case for which large samples are needed. The main aim was to investigate the multilocus genetic distances among the populations (F_ST_), overall and between pairs of populations, and to put this in relation to the migration rates, with the genetic distance among the host populations, and with the geographic distance among the sampling sites. In this regard, it should be noted that lower sample size are needed and that the reliability of the F_ST_ estimates not only depends on the number of individuals per population, but also on the number of sampled populations and the number of markers.

The comparison of the host and pathogen population structures suggests that the organisation of the genetic diversity in the *Ptt* pathogen mirrors that observed for the barley host; this is the case for both the within and between population components of the genetic diversity. In contrast, no match was apparent between the barley and the *Ptm* pathogen. Furthermore, as the *Ptt* population structure is more strongly associated with the barley population structure than with the geographic distances, this suggests that host-mediated gene flow is more important than gene flow along the direct-line distance. This suggests that the barley and the *Ptt* pathogen have similar population dynamics (e.g., drift or bottleneck) and/or patterns of gene flow. Moreover, the observed pattern might at least in part also reflect the reciprocal selection pressures. Indeed, the *Ptt* population structure better correlates with the host population structure at a single SSR locus that is linked to a *Ptt* resistance major QTL, than with the multilocus population structure depicted by the anonymous SSAP markers. Taken together, this suggests that the barley and the *Ptt* pathogen are involved in a co-evolutionary relationship.

If co-evolution is indeed taking place, with the evolutionary rate of a pathogen usually faster than that of its host , the usual expectation is that the pathogen is locally adapted. These arguments apply also to our system here, where the host is strictly selfing^19^ and has only one generation per year; in contrast, the pathogen can undergo both sexual reproduction and clonal reproduction, with many generations during the host cropping cycle^35^. However, a large body of literature has shown that the population structure is also crucial in the determination of co-evolutionary trajectories. Single experimental studies, and simulation studies and meta-analyses have all emphasised the link between evolutionary potential, gene flow, and adaptation^5^,^6^,^7^. In particular, models and simulations show that in the meta-population context, the distribution of resistance and virulence and the detection of adaptive changes will depend on the ratio of the host and pathogen migration rates^5^. When pathogens migrate more than the host (m_p_ > m_h_), it is expected that the pathogen will be locally adapted. When the pathogen migrates at a level similar to its host (m_p_ ≈ m_h_), it will be expected that the relative evolutionary potential is not influenced by migration^5^. When a pathogen migrates less than their host (m_p_ < m_h_), it is possible that gene flow can equalise, or even reverse, the asymmetry in the evolutionary rates between the host and the pathogen; i.e., pathogens might have a co-evolutionary disadvantage and be less able to infect sympatric hosts than allopatric hosts^5^. In our context, local adapted (maladapted) parasites are more (less) able to infect host individuals from their local population. Local adapted (maladapted) host are more (less) resistant to pathogen individuals from their local population^1^.

In our system, the host migrates mainly by seed exchange among farmers^15^,^22^, whereas the pathogen migrates by infected seeds, and by wind and water splashing^35^. Albeit we did not see sharp differences, the three organisms investigated here showed different levels of average population subdivision (F_ST_), which translate into different average rates of migration. We have here inferred more restricted gene flow of the *Ptt* pathogen than the *Ptm* pathogen (m_Ptt_ < m_Ptm_). The moderately high population structure observed for Ptt in this system is in line with other estimates^36^,^37^,^38^, as also the difference in the population structure between Ptt and Ptm^38^,^39^. We also observed a higher level of gene flow of the *Ptm* pathogen compared to the barley host (m_Ptm_ > m_h_), and a lower or similar level of gene flow of the *Ptt* pathogen compared to the host (m_Ptt_ ≤ m_h_). Thus, based on host/pathogen co-evolutionary models^5^, we would expect that in our system the *Ptm* will be locally adapted, and/or that the *Ptt* will be locally maladapted (m_Ptt_ ≤ m_h_) or not involved in a co-evolving interaction (m_Ptt_ = m_h_). Inferring migration from population structures relies on a series of assumption that are unlikely to be met in real populations^40^,^41^. However, this has been applied in several host/pathogen systems and it has allowed comprehensive and very meaningful meta-analyses that have been aimed at the deciphering of the connections between migration and local adaptation^7^.

In concluding here, the correlation between the host and pathogen population structures suggests that local adaption is more likely for the barley—*Ptt* than for the barley—*Ptm* interactions. Moreover, the differences in the migration rates among these organisms further suggest that the interaction of the barley with these two pathogens will have followed different co-evolutionary trajectories.

Detached leaf assays conducted on the first leaf have allowed the identification of international standard set of barley differential genotypes for *P. teres*^42^, as also the screening of a large germplasm collection^43^, the investigation of a gene-for-gene model in a P. teres/barley pathosystem^44^, and the mapping of resistance QTLs using multiple host populations and multiple isolates^45^,^46^. We also used this method to investigate local adaptation in our system.

When analysing data from the cross inoculation test and testing for effects at the pathogen-form level, we observed that the *Ptm* induced necrotic lesions that are significantly larger (+16.84%) than those of the *Ptt*. The rapid spread of the *Ptm* in several parts of the world appears to be a more recent phenomenon, and the reasons for this are not clear^31^. However, if the *Ptm* arrived in Sardinia more recently than the *Ptt*, i.e., if the Sardinian barley evolved without (or with limited) selective pressure exerted by the *Ptm*, it would be expected that the *Ptm* pathogen is more virulent on the Sardinian barley than the *Ptt* pathogen, which is indeed what we have observed. This equal testing of whether the metapopulation of Sardinian barley landraces is ‘better-infected’ by a resident (*Ptt*) than a non-resident (*Ptm*) pathogen thus inferring host adaptation to the *Ptt*. However, such a conclusion might be hampered by differences in the pathogen-form quality and not by host fitness variations.

Furthermore, we observed that the *Ptt* and *Ptm* forms of the pathogen were non-randomly distributed across the six barley fields, and that there was a pathogen form × host population interaction. This all suggests that different demes have experienced different selective histories against the *Ptt* and the *Ptm*.

When testing for effects at within-form local adaptation, a latitudinal cline of host resistance was observed for both the *Ptt* and the *Ptm*, with higher levels of resistance in the north than in the south of the island. *Pyrenophora teres* prefers moist and cold conditions^31^, and these are more likely in the north than in the south of the island (http://www.sar.sardegna.it/). Thus, the pathogen might have exerted differential selective pressures on the host populations at different sites. As the resistance genes in barley against the *Ptt* and *Ptm* are genetically independent (see table 1 of^31^), this suggests that the observed pattern is due at least in part to parallel selection, rather than to a migration/drift process. Interestingly, selection gradients that are oriented along the north-to-south axis in Sardinia were also suggested for the barley host^15^,^22^,^23^.

The main result from the cross-inoculation design is that two closely related pathogens with very similar niches of specialisation and life-style^31^ can give rise to different co-evolutionary outcomes on the same host. This is demonstrated by the ANOVA based on the infection data, where the *Ptt* of the pathogen was shown to be locally adapted, while for the *Ptm* there were no significant plant × pathogen population interactions. Moreover, we also provide evidences that a major resistance QTL on barley chromosome 6H is likely to be involved in the barley—*Ptt* co-evolutionary relationship.

Our cross inoculation experiment was conducted only once. While several informative studies have been conducted as only a single entire cross-inoculation experiment e.g. (12,47), we acknowledge that the repetition of the entire experiment would be of some benefit to reinforce our conclusion. However, it is relevant to note that the detached leaf assay used in this experiment has allowed the mapping of the same QTLs in different genetic backgrounds by different authors and in different laboratories even by conducting the overall experiment only once^45^,^46^. This points towards a good repeatability of our experiment.

Our experiment comprises a relatively low number of isolates per population. However, as shown by Blanquart *et al.*^13^ by simulations, when studying local adaptation, it is better to have a small number of individuals from several populations, instead of sampling many individuals from only a small number of populations, and that at the extreme, it is better to have a lot of populations and only one isolate per population 13. Overall, we considered six fields where the barley and pathogens were sampled. This is an appropriate number of fields^13^, and most importantly, these represent a high fraction of the existing fields where Sardinian barley landraces and the relative infecting *P. teres* pathogens can be sampled. This is relevant, as the estimated local adaptation will also be closer to the true local adaptation of the whole metapopulation^13^.

Moreover, as we adopted the same experimental design for the two forms, if our experiment has low power due to the low number of isolates per population, we would expect that local adaptation will not be detected either for *Ptt* or *Ptm*. However, we observed local adaptation for *Ptt* and not for *Ptm*. Thus, we can assume that local adaptation is indeed present in the *Ptt* form and not in the *Ptm* form, or alternatively, that local adaptation is present in both forms, but the magnitudes of the effects are very different between the two forms: strong in *Ptt* and weak in *Ptm*, such that only in the first case was this detectable in our experiments. Moreover, even if we cannot completely exclude that with more isolate per population the observed scenario would change for some of the details, when the population structures of the two forms were compared, numerous pieces of evidence combined to suggest that the hypothesis that the signal of local adaption found for the *Ptt* form and the contrasting results obtained for *Ptt* and *Ptm* with the cross-inoculation tests might be due to insufficient isolates sampling, is not parsimonious.

Interesting considerations emerged with the comparison of the results of the population structure and the cross-inoculation study. Indeed, the parallelism between the host and pathogen population structures correctly suggested a co-evolving interaction between the barley and the *Ptt* form. This has also been observed in contexts different from that of a landrace metapopulation^8^. Moreover, based on differential migration rates, we would expect local adaptation for the *Ptm* and/or maladaptation (or no signal of adaptation) for the *Ptt*, while the cross-inoculation revealed an almost opposite pattern; i.e., local adaptation for the *Ptt*, and no signal of co-evolving interactions for the *Ptm*.

The absence of a signal for local adaptation for the *Ptm* is in counter tendency compared to the results of a meta-analysis on pathogen local adaptation^7^ that supports the concept that pathogen populations with high relative migration rates are more likely to be adapted to infection of their local host. A possible explanation for this is that in the present study, the geographical scale is not as needed (e.g., it is too small or too large) to detect local adaptation for the *Ptm*, but it is adequate for such for the *Ptt*^4^. Indeed, local adaptation can be detected at the scale of the individual, population or region, depending on the properties and dynamics of any given system^42^. If the pathogen migration is high between the populations (as appears to be the case for the *Ptm* form in our system) and the host populations are characterised by varying frequencies of different resistance types, then it would possible for the pathogen to adapt to the overall metapopulation frequency of the particular host genotypes^4^. In this case, local adaptation might be apparent when testing parasite performance at the local vs. foreign local metapopulation (i.e., at a higher geographic scale). Our study does not specifically show this point. However, as also noted above, the differences in necrosis size between the two forms at the metapopulation levels, as bigger for *Ptm* than for *Ptt*, suggests that at the whole metapopulation level, the *Ptm* might be better adapted than the *Ptm*, although this remains to be investigated in a specific study.

From another side, the same meta-analysis showed that when pathogens have a migration rate lower than that of their hosts, there is not a higher probability that pathogens perform worse on their local host than on allopatric hosts^7^.

Nonetheless, Kaltz *et al.*^48^ suggested that low pathogen migration relative to host migration might account for local maladaptation in a smut fungus—plant system^11^. In this case, the pathogen populations were 12-fold more differentiated that those of its host^11^ (i.e., the difference in the population structure between the host and pathogen were about six-fold that observed in the case of the barley—*Ptt* interaction). Moreover, in their system, the host was an obligate outcrosser, while the pathogen self-fertilises. In this regard, the present system shows a reversed situation, as the barley is a strictly selfing species while the pathogen can undergo both sexual and clonal reproduction.

In our system, the host and pathogens also have very different generation times, with barley undergoing one generation per year, during which time *P. teres* can undergo several cycles^31^. Moreover, the pathogen here can survive on the barley staples where sexual structures (asci) can also be formed^31^; thus, infection will usually be reinitiated by the sexual products of a resident population. Overall, this suggests that factors other than migration must be taken into account here; i.e., that the higher migration rates of the host compared to the *Ptt* pathogen might not be sufficient to reverse the evolutionary advantage due to differences in lifestyle among the two antagonists.

More in particular, based on the correlation analysis, we observed that for explaining the pathogen local adaptation, the host population structure for a locus linked to a QTL for *Ptt* resistance is more important than the overall SSAP structure and the geographic distances. Thus, locus-specific effects are stronger than the ‘neutral’ baseline (represented by the neutral SSAP structure) and the isolation by distance. All of this suggests a role for selection. If in a deme the host resistance genes are selecting pathogens and the pathogen population is highly variable for virulence, it can be expected that the selection will be more efficient when it only acts on the individual of the population; i.e., without the migration that incorporates new genotypes from the surrounding populations. Indeed, in this case, successful pathogens will be diluted together with other (perhaps also less virulent) foreign pathogens, which will reduce the gain of selection. This might be important for *P. teres*, as a typical staples-born pathogen where infections are usually reinitiated by the sexual products of a resident population, and where secondary clonal reproduction can ‘amplify’ a successful pathogen genotype in the population. This would explain why the local adaptation of the pathogen (i.e., barley maladaptation) is high when the pathogen population divergence is high (i.e., when migration is low) also relative to the host. Moreover, this might be even more important when the pathogen has a relatively high virulence, a case where it is possible to observe local adaptation of the pathogen even in the face of a low relative migration rate^6^.

To correlate the differences in the local adaptation patterns observed between the *Ptt* and *Ptm* with differences in lifestyle is not easy. Indeed, both the *Ptt* and *Ptm* forms have very similar lifestyles, and both can undergo sexual reproduction and are staple-borne pathogens. Interestingly, Lightfoot and Able^49^ differentiated the *Ptt* and *Ptm* on the basis of infection characteristics. They observed that while the *Ptt* mainly grows only intercellularly and can affect cells that are not immediately associated with the mycelia, in contrast, the *Ptm* initially forms haustorial-like intracellular vesicles. On this basis, the *Ptt* would be considered as a necrotroph, while the *Ptm* would be a hemibiotroph. However, it is not clear how (and if) this difference might lead to different co-evolutionary outcomes. Moreover, Lajenesse and Forbes^50^ showed that studies based on broad host-range pathogens are less likely to demonstrate local pathogen adaptation. They argued that “this may relate to evolutionary lags during diffuse co-evolution of broad host-range pathogens with their hosts”. This might suggest that the *Ptm* of *P. teres* would have a broader host range than the *Ptt*. However, while comprehensive studies on the host range of *Ptt* have been performed, little information is available on the host range of *Ptm*^31^,^35^. Moreover, the Sardinian populations of barley landraces are cultivated often in sympatry with bred barley cultivars. Moreover, as pointed out by Brown^51^, “crucial in the co-evolutionary dynamic is whether the islands of landrace amid a sea of the same species act as an alternate host with a particular resistance structure or in rotation with bred cultivars of the same species in the same fields” as “the pathogen populations would be subjected to diversifying selection on the alternative populations of the host”. This appears to be important also for *P. teres*, and in particular for the *Ptm*, where epidemics in several parts of the world have been correlated to the large diffusion of susceptible commercial varieties^31^.

The populations used in this study were collected almost 15 years ago; however, the conclusions of our study remain valid: i.e., that coevolution in a landrace metapopulation at this geographical scale is possible, that the evolutionary potential of *P. teres* f. *teres* is high, and that at a given time point the outcomes of the co-evolutionary dynamics can be different for two closely related pathogens occupying the same niche of specialisation. We cannot affirm that repeating the same analyses today with new plant and pathogen populations will lead to the same results. However, this would not indicate that our results are not repeatable, but instead that the populations of both players have changed in number and/or quality during this period of time. However, while we acknowledge that this might be very interesting, our study was not designed to accomplish an over-time analysis of the plant and pathogen relationships.

Concluding, our results have relevant implications for the on-farm conservation of crop resources, plant breeding and deployment of resistance genes.

Most of the interest in on-farm conservation of crop biodiversity is due to the claim that, contrary to the *ex situ* conservation strategy, this provide the opportunity for plants and pathogens to coevolve. Our data suggest that this is indeed the case. However, our results also support the view that the products of coevolutionary dynamics not necessarily encounter the breeders needs and support the argument that the “presence of resistance genes in landraces ‘unfrozen’ on farm will inevitably evoke changes in the pathogen populations that could render the resistances obsolete”^51^. In parallel, our experiment outlines the high evolutionary potential of *P. teres* and suggests that an effective breeding strategy against *P. teres* should focus on quantitative resistance (instead of major resistance) and that, if quantitative resistance are not available, the major resistance genes can better be deployed in rotation trough time or space^52^.

## Materials and Methods

All of the observations in the present study are from six sites in different areas on the island of Sardinia (Italy). Each of these sites corresponded to a field of a highly diverse multigenotypic population of the *S’orgiu sardu* barley landrace^24^, where both the plant (*H. vulgare*) and pathogen (*P. teres*) were sampled (Table 6).

### Host and pathogen sampling

#### The host: Hordeum vulgare

The six host populations of ‘*S’orgiu sardu*’ were sampled in 1999 with the harvesting of 100 spikes along a diagonal in each field^15^. The fields were of similar areas (2–3 hectares). Based on information from the respective farmers, the fields that were chosen for the sampling had been cultivated with each population on the same site for at least 30 years. About 30 lines per population were used for the SSAP and SSR molecular marker characterisation, and six lines per population were used in the cross-inoculation design (Table 6).

#### The pathogen: Pyrenophora teres

Leaves infected with *P. teres* were collected from the same six *H. vulgare* fields between January and April 2000 (Table 6). Infected leaves were collected from randomly selected, non-contiguous plants (at about 10 m apart) along the diagonals of the fields. Single monoconidial isolates were obtained from single lesions from each leaf. Each isolate was designated as *P. teres* f. *teres* (net form, *Ptt*) or *P. teres* f. *maculata* (spot form, *Ptm*) based on the morphology of the original lesion, on the AFLP analysis and the controlled re-inoculation tests^28^, and on the DNA sequence phylogeny^29^. A total of 150 isolates (68 *Ptt*, 82 *Ptm*) were considered here for the population structure analysis, and 34 (17 *Ptt*, 17 *Ptm*) for the cross-inoculation study (Table 6).

**Figure 9:**
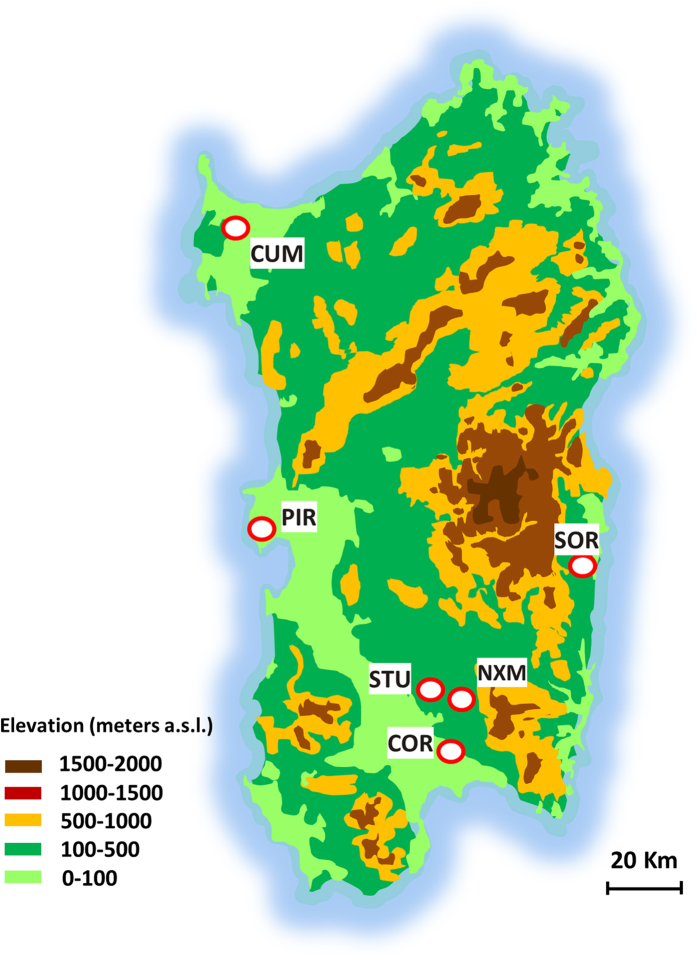
Map of the island of Sardinia, showing the six sites where the plants and pathogen samples were collected. Altimetric information is represented in a simplified way; the map was drawn by the first author of the article (D. Rau) using the drawing tools of Microsoft PowerPoint 2010.

For clarity and brevity the plant and the pathogen samples collected from the same field are labelled with the same name (that of the collection site). The distribution of the collecting sites across the regional territory is shown in Fig. 9.

### Molecular analysis

#### Hordeum vulgare

To characterise the host plants, we used the SSAP markers also used for a study of the population structure and linkage disequilibrium of a larger set of Sardinian barley landrace populations^15^. Each barley line was analysed using six SSAP primer combinations^15^. The SSAP method^53^ exploits the combination of a primer designed on the long terminal repeat sequence of a barley retrotransposon (e.g., Sukkula, Nikita, BAGY-2, BARE-1) and an Mse primer, which usually generates high levels of polymorphism. For further details, the reader is recommended to refer to^15^.

To further characterize host plants, we considered the genotype data obtained by Bellucci *et al.*^23^ at a microsatellite locus (Bmag0009), which is associated (at a distance <0.7 cM) to a major *quantitative trait locus (*QTL) on chromosome 6H that confers resistance against *Ptt*^31^,^32^,^33^,^34^. DNA extraction and SSR genotyping were performed as described by Bellucci *et al.*^23^.

#### Pyrenophora teres

The field isolates were genotyped using AFLPs^54^, as described elsewhere^28^,^30^. Rau *et al.*^28^ reported the data for two primer combinations, using two *Eco*RI (E) primers with two selective nucleotides (E-AC, E-GC) and 1 *Mse*I (M) primer with a selective nucleotide (M-C). Rau *et al.*^30^ extended the analysis, with data from two other primer combinations (E-AC/M-A, E-GC/M-A), to determine whether the isolates studied by Rau *et al.*^30^ that shared the same multilocus genotype are true clones, and to better infer phylogenetic relationships among the isolates^29^. Here, for all of the calculations, we used data from all four primer combinations.

### Cross-inoculation design

The same set of 36 barley lines (belonging to six populations, with six plants per population) was used with both *formae* of the pathogen (Table 6). With the *Ptt*, there were five pathogen populations (CUM, PIR, SOR, NXM, STU) with 1 to 6 isolates per pathogen population, for a total of 17 *Ptt* isolates. For one of the sites (COR), only barley plants and no *Ptt* pathogens were available (Table 6). With the *Ptm form*, there were also five pathogen populations (CUM, PIR, SOR, NXM, COR) with 1 to 4 isolates per pathogen population, for a total of 17 *Ptm* isolates. For the site STU, no *Ptm* isolates were found (see Table 4 of^28^; and Table 6). Thus, the *Ptt* and the *Ptm* pathogens were fully comparable for four barley populations (CUM, PIR, SOR, NXM).

Cross-inoculation tests were performed using a detached leaf assay^42^,^44^ with some modifications Barley seeds were put in plateaux that were kept in a growth chamber under controlled conditions (temperature, 16 °C; relative humidity, 70%). Ten-day-old seedlings were then used. The first leaf of each seedling was cut and segments of 2.0 cm to 2.5 cm in length were placed in Petri dishes on a 0.5-mm layer of 1.2% w/v water agar (Agar Technical N° 3, Oxoid). A single drop (0.1 ml) of a suspension of each monoconidial isolate was put in the centre of each leaf segment. The suspensions were adjusted to 1,000 infective unit/ml^28^,^55^.

**Figure 10:**
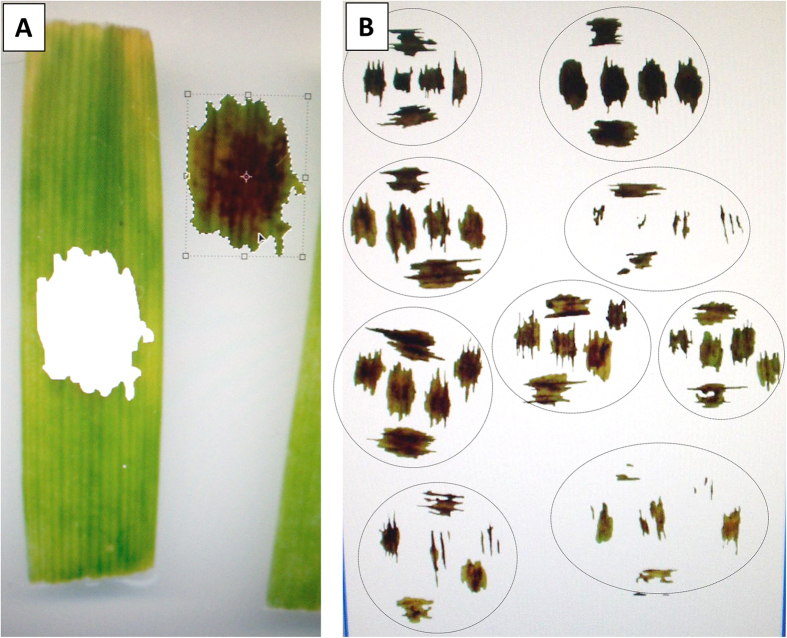
Determination of the area of the necrotic lesions produced by *P. teres* on the barley leaves. (**A**) Each necrotic lesion was cut and (**B**) a collection of necrotic lesions was obtained. As an example, each circle in (**B**) includes the necrotic lesion collected for a host and pathogen combination; nine combinations are represented.

The experimental layout was a completely randomised design with six replicates (i.e., six leaf segments for each line × isolate combination were put in six different Petri dishes and inoculated). The experiments were conducted only once. The Petri dishes were then incubated under 12-h day and 12-h night, at a relative humidity of 80%, for 7 days. To score the infection responses, a semi-automated method was adopted. First, each Petri dish was scanned, to produce an image at a resolution of 600 dpi, which was then imported into a Kodak 1D software scientific image system v. 3.6.1. For each leaf segment, the necrotic area was delimited using the drawing instruments and ‘excised’ using the ‘cut’ options of the software (Fig. 10A,B). The necrotic area was then measured directly by counting the number of pixels in the excised image using the ROI function of the software.

The size of the necrotic lesion was adopted as the measure of fitness for both the host and the pathogen, as microscopic studies have shown that the growth of the pathogen after penetration is significantly slower in resistant host tissue^56^. This trait has also been successfully used to select barley differential lines and to map quantitative resistance loci^43^,^45^,^46^.

### Statistical analysis

#### Molecular analysis

To disentangle the effects of stochastic and demographic processes (mutation, drift, migration) from those putatively due to selection, we searched for loci that showed signatures of selection in the Sardinian landrace SSAP datasets of 189 individuals and 134 polymorphic loci. We used the ‘detection of loci under selection’ from the F-statistic procedure implemented in Arlequin, version 3.5^57^. This is essentially the FDIST approach^58^. We set 10^6^ simulations to build the neutral expectations. Based on the results of this analysis, the full dataset was split into a putatively ‘neutral’ dataset (discarding loci identified as putatively under divergent selection; P < 0.05) and a putatively ‘non-neutral’ dataset (retaining only the loci for which P < 0.05). The statistical analyses were repeated using the three datasets (full, neutral and non-neutral).

Within-population genetic diversity. The within-population genetic diversity was calculated as the average gene diversity (H_E_) across loci using the Arlequin version 3.5 software. The significance of the Pearson’s r coefficients (r) between the within-population genetic diversity statistics of the host and the pathogen were calculated according to the Spearman’s ‘Rho’ non-parametric method, using the JMP software version 7 (Sas Insitute, 2007).

Between-populations genetic differentiation. Population divergence estimates were obtained through the calculation of the Wright’s F-statistics, or F_ST_^59^. For both the plant and pathogen populations, the total variance was partitioned into two levels, as among individuals within populations and among populations, using the hierarchical analysis of molecular variance (AMOVA) implemented in the Arlequin version 3. 5 software. The F_ST_ was determined as the ratio between the variance among the populations and the total variance. The F_ST_ were calculated separately for the *Ptt* and the *Ptm* populations of the pathogen. The significance of all of the F_ST_ estimates was tested by permutations (10^5^ replicates), and the F_ST_ 95% confidence intervals were calculated by bootstrapping over loci with 10^5^ replicates.

To determine whether the different organisms were characterised by populations with different average levels of population subdivision (F_ST_), we also performed analysis of variance according to the Wilcoxon non-parametric test, using the JMP version 7. We estimated the average level of gene flow among populations using based on Wright^59^, which showed that for a haploid organism and for neutral alleles, F_ST_ = 1/(1 + 2 Nm), from which Nm_isl_ = (1-F_ST_)/2 Nm, where N is the local population size and m is the average rate of immigration in an ‘island’ model of population structure.

Geographic distances (km) were calculated on the assumption that the dispersal between populations occurred via a straight line^10^. Partial correlation analysis was performed to examine the relationships for the pathogen genetic distance (as F_ST_) *versus* 1. geography (as km) without the confounding influence of the host, or 2. genetic distance between host populations (F_ST_) without the confounding influence of the geography^10^,^60^. The significance of the standardised correlation coefficients (r) calculated was tested by adopting three-way Mantel tests with permutations (100,000 replicates) using the Arlequin version 3. 5 software.

The differences across the different barley fields in the ratio between the two *formae speciales* of the pathogen were tested using the Fisher exact test, as implemented in the JMP ver. 7 software (Sas Institute, 2007).

#### Cross-inoculation design

As suggested by Blanquart *et al.*^13^, to test for local adaptation we used the following linear model with three factors: host population, pathogen population, and Sympatric *versus* Allopatric plant and pathogen population combinations. Thus, we tested the null-hypothesis that there is no fitness advantage of being in sympatry relative to allopatry. This test is more powerful and easier to interpret than those based on Home *versus* Away and Local *versus* Foreign comparison because it takes into account the variance explained by pathogen and host populations^13^.

Specifically, we adopted the model proposed by Blanquart *et al.*^13^:


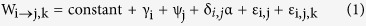


where W_i→j_,_k_ is the performance of the individual host plant *k* of population *i* inoculated with pathogens from population *j*; γ_i_ is the effect of barley population *i*, ψ_j_ is the effect of pathogen population *j*, δ_i,j_ is a binary variable that is 1.0 if *i* = *j* and 0.0 if *i* ≠ *j*, α measures the Sympatric versus Allopatric effect, ε_i,j_ represents the Genotype × Environment (GxE) interaction at the level of the population (with variance σ^2^_ε,pop_), and ε_i,j,k_ that accounts for individual-level error (with variance σ^2^_ε,ind_)^13^.

We tested α against the remainder of the interaction (the ε_*i,j*_), and not against the individual error (the ε_i,j,k_). Indeed, as also pointed out by Blanquart *et al.*^13^ when testing for local adaptation the appropriate unit of replication is the population not the individual. We determined the level of significance of the F statistics for the Sympatric *versus* Allopatric test i with [1, P^2^-2(P-1)-2] degrees of freedom, where P is the number of populations considered in the experiment^13^.

To normalise the distributions of the residuals, the pathogen aggressivity scores were square-root transformed^8^. To determine the ‘weight’ of each population in the meta-population, the same ANOVA model described above was repeated, by jack-knifing over populations; i.e., by sequentially eliminating one population from the dataset.

All of the ANOVAs were performed using JMP version 7.

## Additional Information

**How to cite this article**: Rau, D. *et al.* Co-evolution in a landrace meta-population: two closely related pathogens interacting with the same host can lead to different adaptive outcomes. *Sci. Rep.* **5**, 12834; doi: 10.1038/srep12834 (2015).

## Supplementary Material

Supplementary Information

## Figures and Tables

**Table 1:** Results of the two-way and three-way Mantel tests that show correlations (*r*) and partial correlations between the genetic distances among the pathogen populations *versus* the host population genetic distances and geographical distances among sites.

Datasets and variables		Correlation (r)	P	Partial correlation	P	Explained variance (%)
All markers						
* Ptt* pathogen *vs.*	Barley host	0.586	**0.0107**	0.597	**0.0084**	25.8
	Geography	0.700	0.0725	0.707	0.0592	41.4
* *Barley *vs.*	Geography	0.246	0.6134			
* Ptm* pathogen *vs.*	Barley host	−0.079	0.5770	−0.186	0.6381	1.59
	Geography	0.200	0.3060	0.260	0.2162	5.75
* *Barley *vs.*	Geography	0.431	0.2556			
Putatively neutral						
* Ptt* pathogen *vs.*	Barley host	0.584	**0.0297**	0.543	0.0570	25.3
	Geography	0.644	0.0778	0.611	0.0756	33.3
* *Barley *vs.*	Geography	0.289	0.6301			
* Ptm* pathogen *vs.*	Barley host	0.258	0.3008	0.312	0.2082	9.31
	Geography	−0.022	0.5439	−0.183	0.6267	0.05
* *Barley *v*s.	Geography	0.431	0.2501			
Putatively under selection						
* Ptt* pathogen *vs.*	Barley host	0.414	**0.0041**	0.443	**0.0028**	14.7
	Geography	0.603	0.0555	0.618	0.0555	34.7
* *Barley *vs.*	Geography	0.246	0.6230			
* Ptm* pathogen *vs.*	Barley host	−0.051	0.4681	−0.021	0.4333	0.10
	Geography	−0.299	0.7171	−0.296	0.7173	8.90
* *Barley *vs.*	Geography	0.101	0.5703			

The significance of r was tested using 100,000 permutations. **Bold**, significant correlations. Data are presented for the *Ptt* and *Ptm* forms of the pathogen separately, and using all of the SSAP markers, and only the putatively neutral and putatively under divergence selection markers.

**Table 2:** Mean population divergence estimates (F_ST_) for the barley host (SSAP) and for the *Ptt* and *Ptm* pathogen (AFLP) populations.

Dataset	Organism	Mean F_ST_(1)	95% confidence interval	Nm_isl_
All markers	*Ptt*	0.276 (a)	0.201–0.351	1.312
	Barley	0.189 (b)	0.156–0.223	2.146
	*Ptm*	0.071 (c)	0.047–0.095	6.542
‘Neutral’	*Ptt*	0.221 (a)	0.156–0.292	1.76
	Barley	0.157 (b)	0.133–0.181	2.68
	*Ptm*	0.059 (c)	0.039–0.078	7.97

Letters between parentheses indicate F_ST_ means that are separated with P < 0.05 based on the Tukey-Kramer tests (for this calculation, the negative F_ST_ were set to zero). The F_ST_ 95% confidence intervals are from the bootstrap over the loci (10,000 replications). Nm_isl_ = migration rate assuming an island model of population structure.

^(1)^All F_ST_ were significant at the permutation tests, with P between <10^−3^ and <10^−5^.

**Table 3:** ANOVA illustrating the effects of the *Ptt* and *Ptm* pathogens, the barley host population, and the *forma specialis* × host population on necrosis size. d.f. = degrees of freedom; SS = sum of squares; F = F ratio.

Source	d.f.	SS	F ratio	Probability
*Forma specialis*	1	209885.2	127.5	<0.0001
Host population	5	612920.5	74.5	<0.0001
*Forma* × host population	5	79578.2	9.7	<0.0001
Error	6715	11052804	1646	

**Table 4:** Results of the analysis of variance of the infection data for the *Ptt* and *Ptm* separately.

Source	*Ptt*				*Ptm*			
	*d.f.*	SS	F	Probability	*d.f.*	SS	F	Probability
Host pop(1)	4	193309.0	25.08	<0.0001	4	210156.2	62.65	<0.0001
Pathogen pop(1)	4	1106076.7	143.51	<0.0001	4	580584.3	173.07	<0.0001
Sympatric *versus* allopatric(2)	1	19885.6	5.62	0.0285	1	8.8	0.01	0.9300
Host pop. xpath. pop. remainder(1)	19	67255.0	1.84	0.0147	19	19650.9	1.23	0.2198
Error	3362	6477994.9			3305	2771770.9		

For this analysis, all of the host populations were retained; i.e., for both the *Ptt* and the *Ptm* of the pathogen, we considered six host populations. For the *Ptt*, the host population COR do not have the corresponding pathogen population, while for the *Ptm* the host population STU do not have the corresponding pathogen population. d.f. = degrees of freedom; SS = sum of squares; F = F ratio.

^(1)^Tested for error variance.

^(2)^Tested for the remainder of the host population × the remainder of the pathogen population.

**Table 5:** Results of the jack-knifing over the populations.

Eliminated population	Sympatric *versus* Allopatric	
	F	P
CUM	1.92	0.194
STU	2.81	0.122
PIR	5.01	0.047
TER	5.42	0.040
NXM	5.68	0.036

The same model as in Table 5 was applied while sequentially eliminating each host population, to determine the P value for the Sympatric *versus* Allopatric term for the *Ptt* pathogen. This analysis was performed considering all six of the host populations (i.e., including COR, for which no pathogen sample was available). The populations are in descending order based on their ‘weight’ on the P value for the Sympatric *versus* Allopatric term.

**Table 6:** Codes and sample sizes of the host (barley) and pathogen (barley net blotch) samples.

Host population sample(1)			Corresponding pathogen sample(2)		
Code	Number of lines characterised by SSAP(1)	Number of lines in the cross-inoculation (same for Ptt and Ptm)	Code	Number of isolates characterised by AFLP (Ptt, Ptm)(2)	Number of isolates in the cross-inoculation (Ptt, Ptm)
CUM	33	6	SEC	31 (2,29)	2, 4
PIR	33	6	PIR	24 (16,8)	5, 4
SOR	32	6	TER	26 (6,20)	3, 4
NXM	31	6	SIR	21 (3, 18)	1, 4
STU	30	6	BAC	36 (36,0)	6, 0
COR	30	6	SES	12 (5, 7)	0, 1

^(1)^Based on Rodriguez *et al.* 2012^25^.

^(2)^Based on Rau *et al.* 2003^31^.
