# Supplementary material for: Co-evolution in a landrace meta-population: two closely related pathogens interacting with the same host can lead to different adaptive outcomes
Source: Sci Rep. 2015 Aug 7;5:12834. doi: 10.1038/srep12834 (PMC4528193; doi:10.1038/srep12834)
Supplement: Supplementary Information [file srep12834-s1.doc]

Supporting information

**Co-evolution in a landrace meta-population: two closely related pathogens interacting with the same host can lead to different adaptive outcomes**

Domenico Rau, Monica Rodriguez, Maria Leonarda Murgia, Virgilio Balmas, Elena Bitocchi, Elisa Bellucci, Laura Nanni, Giovanna Attene, Roberto Papa

**Figure S1.** Correlation between pathogen aggressivity and latitude. Calculations are for the *NF* (top) and *SF* (bottom) separately.

**Table S1.** Statistics for the distribution of the two *formae speciales* of the pathogen, as *Ptt* and *Ptm*, within each barley field and among the barley fields.

| **Field name** | **All isolates** | ***Ptt* isolates** | ***Ptm* isolates** | ***χ^2^*** | **d.f.** | **P**  **Figure 2** – Correlation (Pearson, *r*) between the genetic diversity of *Pyrenophora teres* pathogen populations (***NF***  and ***SF*** ) and of *Hordeum vulgare* host populations. For barley, three datasets were used: SSR (a), ISSR (b) and combined (ISSR+SSR) (c). *P* = significance level.  * The correlation is significant also after Bonferroni correction (0.05/6 = 0.0083 > P = 0.0002). |
| --- | --- | --- | --- | --- | --- | --- |
| **Within fields** | | | | | | |
| CUM | 31 | 2 | 29 | 23.52 | 1 | 1E-06 |
| PIR | 24 | 16 | 8 | 2.67 | 1 | 0.102 |
| TER | 26 | 6 | 20 | 7.54 | 1 | 0.006 |
| NXM | 21 | 3 | 18 | 10.71 | 1 | 0.001 |
| STU | 36 | 36 | 0 | 36.00 | 1 | 2E-09 |
| COR | 12 | 5 | 7 | 0.33 | 1 | 0.564 |
| **All fields** | 150 | 68 | 82 | 1.31 | 1 | 0.253 |
| **Among fields** |  |  |  | 80.16 | 5 | <0.001 |

*χ^2^*, Chi-squared, based on a 1:1 ratio with one degree of freedom (*d.f.*) and the probability (*P*) of a greater *χ^2^* value under the null hypothesis of the 1:1 ratio*.*

**Table** S2. Results of the analysis of variance of the infection data for the *Ptt* and *Ptm* separately. For this analysis, only the host populations were considered for which there was a corresponding pathogen population; i.e., for the *Ptt*, the host population COR was not considered, while for the *Ptm*, the host population STU was not considered.

| **Source** | ***Ptt*** | | | |  | ***Ptm*** | | | |
| --- | --- | --- | --- | --- | --- | --- | --- | --- | --- |
|  | ***d.f.*** | **SS** | **F** | **Probability** |  | ***d.f.*** | **SS** | **F** | **Probability** |
| Host population^(1)^ | 4 | 193309.0 | 26.16 | <.0001 |  | 4 | 210156.2 | 60.82 | <.0001 |
| Pathogen population ^(1)^ | 4 | 936405.0 | 126.71 | <.0001 |  | 4 | 530420.3 | 153.50 | <.0001 |
| Sympatric *versus* allopatric ^(2)^ | 1 | 19432.0 | 5.36 | 0.0351 |  | 1 | 8.6 | 0.01 | 0.9205 |
| Host pop. × host path remainder ^(1)^ | 15 | 54354.7 | 1.96 | 0.0146 |  | 15 | 15696.0 | 1.21 | 0.2547 |
| Error | 2804 | 5180539.3 |  |  |  | 2823 | 2438733.4 |  |  |

^(1)^, Tested over the error variance

^(2)^, Tested over the remainder of the host population × remainder of the pathogen population

**Table S3.** Results of jack-knifing over the populations. The same model as in Table 5 was applied while sequentially eliminating each host population, to determine the P value for the Sympatric *versus* Allopatric term for the *Ptt* pathogen. This analysis was performed considering five host populations (i.e., excluding COR for which no pathogen sample was available). The populations are in descending order based on their ‘weight’ on the P value for the Sympatric *versus* Allopatric term.

| **Eliminated** | **Sympatric *versus* Allopatric** | |
| --- | --- | --- |
| **population** | **F** | **P** |
| CUM | 1.91 | 0.194 |
| STU | 2.79 | 0.133 |
| PIR | 4.13 | 0.077 |
| TER | 4.77 | 0.061 |
| NXM | 7.51 | 0.025 |

**Table S4**. Pairwise populations of the F_ST_ matrices for the *Ptt* pathogen and for the barley host. In both cases, only putatively neutral markers were used. Underlined, significant comparison (P >0.05).

|  | **CUM** | **PIR** | **SOR** | **NXM** | **STU** |
| --- | --- | --- | --- | --- | --- |
| **NF Pathogen AFLP ‘neutral’ divergence** | | | | | |
| CUM |  |  |  |  |  |
| PIR | 0.332 |  |  |  |  |
| SOR | 0.383 | 0.121 |  |  |  |
| NXM | 0.176 | 0.001 | 0.143 |  |  |
| STU | 0.388 | 0.245 | 0.250 | 0.058 |  |
| **Host SSAP ‘neutral’ divergence** | | | | | |
| CUM |  |  |  |  |  |
| PIR | 0.187 |  |  |  |  |
| SOR | 0.171 | 0.009 |  |  |  |
| NXM | 0.1839 | 0.160 | 0.140 |  |  |
| STU | 0.2455 | 0.226 | 0.203 | 0.092 |  |
